# Tuberculosis Mortality by Occupation in South Africa, 2011–2015

**DOI:** 10.3390/ijerph15122756

**Published:** 2018-12-05

**Authors:** Tahira Kootbodien, Kerry Wilson, Nonhlanhla Tlotleng, Vusi Ntlebi, Felix Made, David Rees, Nisha Naicker

**Affiliations:** 1 National Institute for Occupational Health, National Health Laboratory Service, Braamfontein, Johannesburg 2001, South Africa; kerryw@nioh.ac.za (K.W.); nonhlanhlat@nioh.ac.za (N.T.); vusi.ntlebi@nioh.nhls.ac.za (V.N.); felixm@nioh.ac.za (F.M.); david.rees@nioh.nhls.ac.za (D.R.); nishan@nioh.ac.za (N.N.); 2 School of Public Health, Faculty of Health Sciences, University of the Witwatersrand, Witwatersrand 2193, South Africa; 3 Environmental Health Department, Faculty of Health Sciences, University of Johannesburg, Johannesburg 2028, South Africa

**Keywords:** tuberculosis, occupation, mortality, age-standardised mortality rates

## Abstract

Work-related tuberculosis (TB) remains a public health concern in low- and middle-income countries. The use of vital registration data for monitoring TB deaths by occupation has been unexplored in South Africa. Using underlying cause of death and occupation data for 2011 to 2015 from Statistics South Africa, age-standardised mortality rates (ASMRs) were calculated for all persons of working age (15 to 64 years) by the direct method using the World Health Organization (WHO) standard population. Multivariate logistic regression analysis was performed to calculate mortality odds ratios (MORs) for occupation groups, adjusting for age, sex, year of death, province of death, and smoking status. Of the 221,058 deaths recorded with occupation data, 13% were due to TB. ASMR for TB mortality decreased from 165.9 to 88.8 per 100,000 population from 2011 to 2015. An increased risk of death by TB was observed among elementary occupations: agricultural labourers (MOR_adj_ = 3.58, 95% Confidence Interval (CI) 2.96–4.32), cleaners (MOR_adj_ = 3.44, 95% CI 2.91–4.09), and refuse workers (MOR_adj_ = 3.41, 95% CI 2.88–4.03); among workers exposed to silica dust (MOR_adj_ = 3.37, 95% CI 2.83–4.02); and among skilled agricultural workers (MOR_adj_ = 3.31, 95% CI 2.65–4.19). High-risk TB occupations can be identified from mortality data. Therefore, TB prevention and treatment policies should be prioritised in these occupations.

## 1. Introduction

South Africa is one of 22 high tuberculosis (TB) burden countries identified by the World Health Organization (WHO), with a TB incidence rate of 520 per 100,000 population in 2015 [1]—a decline from a rate of 762 per 100,000 population in 2011 [2]. High rates of human immunodeficiency virus (HIV) infection have been a key driver of TB incidence in South Africa, with a reported estimated HIV prevalence of 18% for adults aged 15 to 49 years [3] and HIV-TB coinfection rates of 340 per 100,000 population [4]. Moreover, there has been a growing burden of multidrug-resistant (MDR) TB (25 per 100,000 population) [4] and the emergence of extensively drug-resistant (XDR) TB in South Africa [5]. 

There has been an increasing awareness of occupation contributing to TB morbidity and mortality. Many studies have reported that the risk of TB is elevated in health care workers exposed to persons with active TB [6,7]; among workers exposed to silica dust [8,9,10]; and workers in occupations associated with lower socioeconomic status, such as migrant farm workers [11,12]. Workplaces are therefore an important risk factor for the transmission of TB in high burden settings. Adequate surveillance of TB by industry or occupation can provide a clearer understanding of the burden and distribution of TB and can identify trends and emerging patterns in workplaces in order to support recommendations for TB prevention programmes. In the US, mortality data as well as data from the Bureau of Labour have been used to estimate the annual burden of occupational disease mortality in order to identify risky occupations and exposed populations [13]. In England and Wales, mortality data of men aged 20 to 74 years were analysed from 1979 to 2010 to monitor trends in mortality related to workplace hazards [14]. Similarly, in the absence of a national occupation surveillance programme in South Africa, our study aims to understand the patterns associated with occupation and TB mortality by using available occupation information from vital registration data as a mortality surveillance tool to identify occupations at increased risk.

## 2. Materials and Methods

### 2.1. Data Sources and Data Management

Underlying cause of death was based on death certificate information reported to Statistics South Africa (StatsSA) for the period of 2011 to 2015 by the Department of Home Affairs [15]. Death certificates are mandatory for all deaths. However, challenges with the quality of cause of death data have been reported, in particular missing information on the death certificates [16] as well as the use of codes for ill-defined and unknown causes of death [17]. Deaths due to TB included underlying cause of death data coded as A15 to A19, B90, J90, J94, U51, and U52 according to the 10th version of the International Classification of Diseases (ICD-10) and grouped according to the second South African National Burden of Disease (NBD) study [18]. The usual occupation of the deceased is recorded on the death certificate and refers to the type of work done by the deceased for most of his or her working life. Usual occupation is then classified into major occupation groups by StatsSA based on the South African Standard Classification of Occupations (SASCO), which consists of 10 major groups and 41 sub-major groups [19]. We used nine of the 10 major groups: managers (SASCO1); professionals (SASCO2); technicians and associate professionals (SASCO3); clerks (SASCO4); service workers, shop and market sales workers (SASCO5); skilled agricultural and fishery worker (SASCO6); craft and related trade workers (SASCO7); plant, machine operators and assemblers (SASCO8); and elementary occupations (SASCO9). Armed forces, occupations unspecified, not elsewhere classified, and not economically active persons (SASCO0) was not included in the occupation group analyses, as it contains unemployed persons. Elementary occupations include occupations which require knowledge and experience of simple and routine tasks which may require the use of handheld tools and physical effort, such as cleaners and helpers and labourers [19]. Sub-groups are sub-divisions of the major occupation groups and are denoted by a two-digit code. Some individuals have occupation sub-group data recorded but not occupation group, which accounts for the larger number with sub-group data presented in the results. 

### 2.2. Statistical Analysis

Age-standardised mortality rates (ASMRs) per 100,000 population were calculated using mid-year population estimates provided by StatsSA and by direct standardisation with the WHO standard population for all South Africans of working age in 2011 to 2015 [20]. Individuals for whom sex or age were unspecified or unknown were not included in the analyses. Occupation data were available for only 16% of all recorded deaths from 2011 to 2015. We could not calculate ASMRs by occupation group because of the low numbers reported for some occupations and information on the number of individuals employed in each occupation group was not available [21]. Thus, multiple logistic regression analysis was performed to identify sub-occupations associated with TB mortality. We tested the association between TB mortality and sociodemographic factors such as age, sex, year of death, province of death, educational attainment, and smoking status. Variables with *p*-values of <0.10 in the univariate analysis were included in the multivariate analysis. Mortality odds ratios (MORs) and 95% confidence intervals (CIs) are reported to indicate the association’s strength and direction.

## 3. Results

### 3.1. TB Mortality 

In total, there were 2,377,676 recorded deaths from 2011 to 2015. Approximately 14% (188,230) of individuals, aged 15 to 64 years, had TB assigned as the underlying cause of death (Table 1). TB deaths decreased from 2011 to 2015 by approximately 10%. Males comprised 59% of all TB deaths, while TB deaths as a percentage of all deaths were highest among persons aged between 35 to 39 years (16%). Those with a level of education between grade 8 and grade 12 (29%) and those who died in KwaZulu-Natal Province (25%) provided the highest percentages of TB deaths by education and province, respectively.

The MORs showed a decreasing trend in TB deaths as a percentage of all deaths from 2011 to 2015, while age groups 30–44 were at most risk of a TB death. Those with a Grade 1–7 (primary school) education were at highest risk of a TB death followed by those with no education. KwaZulu-Natal Province had the highest proportions as well as the highest odds of TB deaths, with a quarter of deaths attributed to TB. Reported smoking resulted in a small significant increase in the odds of TB death.

**Figure 1:**
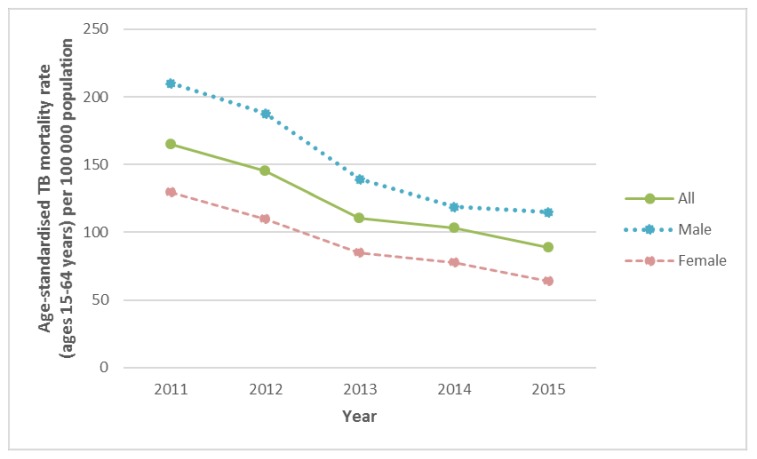
Age-standardised TB mortality rate (ages 15–64 years) per 100,000 population, overall and by sex.

Age-standardised TB mortality rates for persons aged 15 to 64 years are shown in Figure 1. TB mortality substantially decreased from 165.9 to 88.8 per 100,000 population for persons aged 15 to 64 years from 2011 to 2015. The decline was observed in men and women. TB mortality was higher among men than women.

### 3.2. TB Mortality by Occupation Group

**Figure 2:**
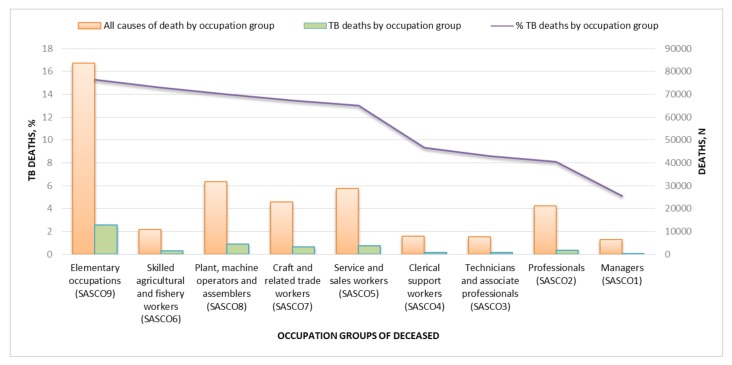
Percentage and number of TB deaths by occupation groups, 2011–2015 (*n* = 221,058).

Occupation group data were available for 221,058 (15.9%) individuals aged 15 to 64 years. Of those with occupation group data, 29,084 (13.2%) individuals had TB recorded as the underlying cause of death: 19,750 (67.9%) males and 9290 (32.1%) females. Elementary occupations (SASCO9) recorded the highest percentage of TB deaths (15.3% of all deaths in the group), followed by skilled agricultural and fishery workers (SASCO6, 14.6%) and plant, machine operators and assemblers (SASCO8, 14.0%) (Figure 2). TB deaths were lowest among managers (SASCO1, 3.1%) and professionals (SASCO2, 4.3%). Plant, machine operators and assemblers worked predominantly (66.9%) in the mining sector.

### 3.3. TB Mortality by Occupation Sub-Group

**Figure 3:**
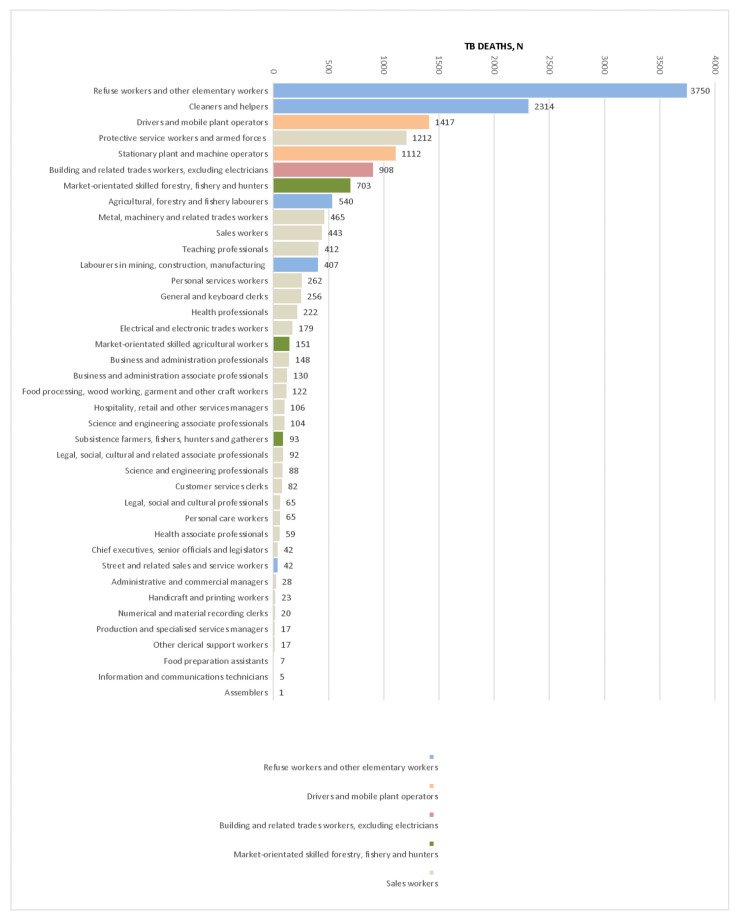
Number of TB deaths by occupation sub-groups.

Occupation sub-group data were available for 312,355 (22.6%) individuals aged between 15 and 64 years for the period 2011 to 2015 (Figure 3). Approximately 10% (31,620) of individuals had TB recorded as the underlying cause of death. TB deaths as a percentage of all deaths in the sub-groups was highest among low socioeconomic occupations sub-groups (i.e., agricultural, forestry and fishery labourers (12.2%), refuse workers (12.0%) and cleaners and helpers (9.14%)). Groups of occupations with potential for high silica exposure and high risk for TB were labourers in mining, construction, and manufacturing (11.7%) and building and related trade workers (11.6%). Occupations associated with potential contact with TB patients had relatively low percentages of TB deaths: health professionals (5.0%) and health associate professionals (6.1%).

Adjusted mortality odds ratios for TB by occupation sub-groups are presented in Table 2. Low socioeconomic occupations had a three-fold increased risk of dying from TB when compared to business and administration professionals: agriculture, forestry, and fisheries labourers (MOR = 3.58, 95% CI 2.96–4.32), cleaner and helpers (MOR = 3.44, 95% CI 2.91–4.09), refuse workers (MOR = 3.41, 95% CI 2.88–4.03), and street workers (MOR = 3.27, 95% CI 2.27–4.69). Similarly, the risk of dying from TB was three times higher among groups of occupations with potential for high silica exposure such as labourers in mining, construction, and manufacturing (MOR = 3.26, 95% CI 2.68–3.96), building and related trades workers (MOR = 3.24, 95% CI 2.71–3.87), and stationary plant and machine operators (MOR = 3.37, 95% CI 2.83–4.02) compared to business and administration professionals. In addition, skilled agricultural workers (MOR = 3.31, 95% CI 2.65–4.19) had a three times greater risk of dying from TB compared to business and administration professionals.

TB deaths were nearly twice as likely among health professionals (MOR = 1.86 95% CI 1.51–2.31) and health associate professionals (MOR = 2.05, 95% CI 1.50–2.80), whereas personal care workers (MOR = 2.99, 95% CI 2.21–4.07) had nearly a three times greater risk compared to business and administration professionals. Higher socioeconomic occupations such as legal, social, and cultural professionals (MOR = 1.24, 95% 0.92–1.67), science and engineering professionals (MOR = 1.21, 95% CI 0.93–1.59), and chief executives and senior officials (MOR = 1.18, 95% CI 0.83–1.68) were not significantly associated with elevated TB mortality compared to business and administration professionals.

## 4. Discussion

TB is the leading cause of death in South Africa, and in 2015, TB accounted for approximately 11% of deaths in persons aged 15 to 64 years [15]. We found substantial differences in TB mortality across occupation groups, which may reflect differences in socioeconomic levels, HIV-burden and other risk factors for TB, and occupational exposure to silica dust. Overall, TB mortality was much higher in lower socioeconomic occupations such as elementary occupations and agricultural and fishery workers than in higher socioeconomic categories such as managers and professionals. Populations working in lower socioeconomic occupations are vulnerable to conditions of poverty, food insecurity, and malnutrition and have barriers to health care access that may increase susceptibility to TB infection and disease severity in high burden TB areas [12]. The association between TB and poverty is mediated by overcrowding and poor ventilated housing, which may increase the risk of TB infection [22,23]. In addition, socioeconomic-associated factors such as tobacco smoking, exposure to second hand smoke, and indoor air pollution from biomass fuels are risk factors for TB disease [24] as well as TB mortality [25].

Over the past few years, the TB incidence rate has fallen by approximately 2% per year worldwide and more rapidly in South Africa, at 7% per year, and this decrease is thought to be due to the expansion of the TB and HIV programmes and the increased coverage of antiretroviral treatment among HIV-positive individuals [4]. We reported a decline in the absolute number of TB deaths in South Africa, which is in keeping with the 42% global reduction in TB mortality from 2010 to 2017 [4]. Nevertheless, TB deaths accounted for just over a quarter of all deaths in 15- to 64-year-olds during 2011–2015 in the province of KwaZulu-Natal. Within South Africa, KwaZulu-Natal Province has the highest prevalence of drug-resistant (MDR and XDR) TB [26], and elevated TB mortality may be explained by the high prevalence of HIV and drug-resistant TB co-infection [27].

We observed elevated odds of TB mortality among agricultural workers (skilled workers as well as labourers) compared to business and administration professionals. Farm workers in South Africa are often migrant or seasonal workers who live in rural communities and experience difficulties in accessing health care services [28]. TB mortality odds were elevated among plant and machine operators, who predominantly worked in the mining sector, compared to those not economically active. Notwithstanding the environmental conditions that are favourable for TB transmission such as enclosed spaces with poor ventilation, many mine workers are migrants who are at increased risk of HIV infection [29]. Moreover, gold miners exposed to silica dust with or without a diagnosis of silicosis also have an increased risk of TB [8,10]. When combined with silicosis, HIV infection multiplicatively increases the risk of TB in miners compared to miners without HIV infection and silicosis [9]. Workers in the building and construction industry are also exposed to high levels of silica dust during activities such as blasting, cutting, and grinding of silica-rich building materials [30,31] and are at increased risk of TB [32].

A two- to three-fold increased TB mortality risk was observed among health professionals, health associate professionals, and personal care workers compared to business professionals. TB is an important occupational hazard among health care workers [33] and the risk of TB infection is higher among health care workers than the general population [34]. In resource-poor settings in low- and middle- income countries, the risk of TB infection is also increased where infection control measures are inadequate [6]. In addition, health care workers in South Africa have an estimated HIV prevalence of approximately 16%, which is close to the general population and among the highest in the world [35]. Recently, a study in KwaZulu-Natal Province reported that health care workers living with HIV (Odds Ratio (OR) = 6.35, 95% CI 3.54–11.37) and those spending time working in areas with TB patients (OR = 2.24, 95% CI 1.40–3.59) were at increased risk of developing TB [36]. Reports have also highlighted a lack of awareness and knowledge of infection control policies by health care workers and poor adherence to TB preventative measures [37,38]. However, health care workers are less likely to have delays in TB diagnosis and treatment and have improved treatment outcomes [39]. In addition, the level of education of health care professionals as well as higher socioeconomic status may have contributed to the relatively low percentage of TB deaths compared to low socioeconomic occupations [12].

The findings in this study are subject to several limitations. The completeness of death registration data and accurate coding of the underlying cause of death are important for mortality surveillance systems. TB is common in South Africa and many doctors are experienced in its diagnosis. Despite this, inaccurate TB diagnoses have been shown in South Africa in living gold miners when compared to their autopsy findings and in populations in other settings [40], so some misclassification of TB is to be expected. However, if misclassification did occur, it is unlikely to have been differentially distributed across occupation groups and sub-groups to an extent sufficient to explain the differences in MORs by occupation. Thus, as TB is common in South Africa and diagnosed easily, misclassification would not be limited to one occupation group only. The small percentage of individuals with occupation information retrieved from the death certificates resulted in approximately 84% of recorded TB deaths with absent occupation information, which may have affected the generalisability of our findings, but we are unable to estimate the effect of these missing data. Underestimation of the South African mid-year population estimates [41] may have overestimated the mortality rates.

## 5. Conclusions

Accurate estimates of, and surveillance for, occupationally associated TB mortality are limited because death certificates lacked comprehensive occupational data. These data are necessary for the appropriate planning and allocation of resources for effective TB prevention and treatment programmes and to promote TB case finding and medical surveillance in high-risk occupations and industries. Nonetheless, we have identified elementary occupations, skilled agriculture and fishery workers, and plant and machine operators as groups at high risk for TB mortality. This study suggests that high-risk TB occupations and industries can be identified from mortality data so that efforts to prevent the disease and diagnose and treat cases can be appropriately focused. Longitudinal studies are therefore needed to identify individual risk factors in these groups at high risk of TB mortality.

## Figures and Tables

**Table 1:** Summary of tuberculosis (TB) deaths among persons aged 15 to 64 years and selected characteristics—South Africa, 2012–2016 (*n* = 188,230).

Characteristic	Total Deaths	Number of TB Deaths	%	Crude MOR ^*^ (95% CI)	*p*-Value
Total number of deaths	2,377,676	217,676	9.15		
Number of deaths (15–64 years)	1,384,609	188,230	13.59		
Year of death					
2011	258,487	48,488	25.76	Reference	
2012	284,951	42,202	22.42	0.93 (0.91–0.94)	<0.001
2013	269,046	35,665	18.95	0.81 (0.80–0.83)	<0.001
2014	261,047	33,082	17.58	0.77 (0.76–0.79)	<0.001
2015	262,590	28,793	15.30	0.65 (0.64–0.67)	<0.001
Age groups					
15–19	35,807	3006	1.60	Reference	
20–24	76,000	8771	4.66	1.42 (1.36–1.49)	<0.001
25–29	129,895	20,012	10.63	1.98 (1.91–2.07)	<0.001
30–34	159,832	27,451	14.58	2.26 (2.17–2.35)	<0.001
35–39	166,105	29,737	15.80	2.38 (2.28–2.47)	<0.001
40–44	160,167	26,915	14.30	2.20 (2.11–2.29)	<0.001
45–49	156,123	23,339	12.40	1.92 (1.84–1.99)	<0.001
50–54	162,768	20,004	10.63	1.53 (1.46–1.59)	<0.001
55–59	164,621	16,026	8.51	1.18 (1.12–1.23)	<0.001
60–64	173,291	12,969	6.89	0.88 (0.84–0.92)	<0.001
Sex					
Female	593,674	77,282	41.06	Reference	
Male	786,621	110,280	58.59	1.09 (1.07–1.10)	<0.001
Unspecified	3583	661	0.35	1.23 (1.13–1.34)	<0.001
Educational attainment					
None	71,596	10,062	5.36	1.85 (1.76–1.94)	<0.001
Primary education	236,807	36,229	19.35	2.04 (1.95–2.14)	<0.001
Secondary education	399,912	54,259	28.93	1.78 (1.69–1.86)	<0.001
Tertiary education	26,607	2161	1.15	Reference	
Unspecified	595,021	78,417	41.81	1.71 (1.64–1.79)	<0.001
Province of death					
KwaZulu-Natal	268,373	47,374	25.17	1.51 (1.47–1.56)	<0.001
Gauteng	292,504	32,326	17.17	0.86 (0.85–0.90)	<0.001
Eastern Cape	195,957	27,559	14.64	1.15 (1.11–1.19)	<0.001
Mpumalanga	112,285	16,903	8.98	1.25 (1.20–1.29)	<0.001
Limpopo	128,223	16,140	8.57	1.01 (0.98–1.05)	<0.001
North West	104,394	14,970	7.95	1.18 (1.14–1.22)	<0.001
Free State	106,102	13,529	7.19	1.03 (0.99–1.07)	<0.001
Western Cape	127 591	13,498	7.17	0.83 (0.81–0.86)	<0.001
Northern Cape	42,221	5242	2.78	Reference	
Unspecified	1889	256	0.14	1.15 (0.97–1.27)	<0.001
Smoking history					
No	513,389	70,830	37.63	Reference	
Yes	284,717	41,320	21.95	1.06 (1.05–1.07)	<0.001
Unknown	72,570	9446	5.34	0.93 (0.91–0.95)	<0.001
Unspecified	506,662	66,040	35.08	0.94 (0.93–0.95)	<0.001

* MOR = Mortality odds ratio.

**Table 2:** Adjusted TB mortality odds ratios (MORs) by occupation sub-groups among persons aged 15–64 years in South Africa, 2011–2015.

Sub-Occupation Groups	TB Deaths	Total Deaths	%	Adjusted MOR *	95% CI	*p*-Value
Business and administration professionals	148	5064	2.92	Reference		
Agricultural, forestry, and fishery labourers	540	4445	12.15	3.58	2.96–4.32	<0.001
Cleaners and helpers	2314	25,313	9.14	3.44	2.91–4.09	<0.001
Refuse workers and other elementary workers	3750	31,250	12.00	3.41	2.88–4.03	<0.001
Market–orientated skilled forestry, fishery, and hunters	703	6147	11.44	3.39	2.83–4.07	<0.001
Stationary plant and machine operators	1112	9412	11.81	3.37	2.83–4.02	<0.001
Market–orientated skilled agricultural workers	151	1283	11.77	3.31	2.65–4.19	<0.001
Street and related sales and service workers	42	392	10.71	3.27	2.27–4.69	<0.001
Labourers in mining, construction, manufacturing	407	3472	11.72	3.26	2.68–3.96	<0.001
Building and related trades workers	908	7835	11.59	3.24	2.71–3.87	<0.001
Personal care workers	65	685	9.49	2.99	2.21–4.07	<0.001
Drivers and mobile plant operators	1417	12,841	11.03	2.89	2.43–3.44	<0.001
Protection service workers and armed forces	1212	11,397	10.63	2.76	2.31–3.29	<0.001
Legal, social, cultural, and related associate professionals	92	1093	8.42	2.74	2.02–3.47	<0.001
Sales workers	443	5119	8.65	2.58	2.13–3.13	<0.001
Food processing, wood working, garment, and other craft workers	122	1619	7.54	2.49	1.09–3.20	<0.001
Personal service workers	262	3838	6.83	2.27	1.84–2.79	<0.001
Customer services clerks	82	1282	6.39	2.13	1.61–2.81	<0.001
Electrical and electronic trades workers	179	2332	7.68	2.12	1.70–2.66	<0.001
Metal, machinery, and related trades workers	465	6218	7.48	2.11	1.74–2.55	<0.001
Health associate professionals	59	971	6.08	2.05	1.50–2.80	<0.001
Health professionals	222	4452	4.99	1.86	1.51–2.31	<0.001
Teaching professionals	412	8000	5.15	1.76	1.45–2.13	<0.001
Legal, social, and cultural professionals	65	1836	3.54	1.24	0.92–1.67	0.153
Administrative and commercial managers	28	760	3.68	1.23	0.82–1.87	0.316
Science and engineering professionals	88	2361	3.73	1.21	0.93–1.59	0.154
Subsistence farmers, fishers, hunters, and gatherers	93	2665	3.49	1.20	0.92–1.57	0.171
Chief executives, senior officials, and legislators	42	1205	3.49	1.18	0.83–1.68	0.344
Hospitality, retail, and other services managers	106	3471	3.05	0.94	0.73–1.22	0.687

* Adjusted for age, sex, year of death, province of death, and smoking status.
